# Chemical Composition and Insecticidal Activity of Essential Oil from *Coriandrum sativum* Seeds against *Tribolium confusum* and *Callosobruchus maculatus*

**DOI:** 10.5402/2012/263517

**Published:** 2012-11-25

**Authors:** Abbas Khani, Tahere Rahdari

**Affiliations:** Department of Plant Protection, Faculty of Agriculture, University of Zabol, P.O. Box 9861335856, Zabol, Iran

## Abstract

The biological activity of essential oil extracted from coriander, *Coriandrum sativum* L. (Apiaceae), seeds against adults of *Tribolium confusum* Duval (Coleoptera: Tenebrionidae) and *Callosobruchus maculatus* F. (Coleoptera: Bruchidae) was investigated in a series of laboratory experiments. Fumigant toxicity was assessed at 27 ± 1°C and 65 ± 5% R.H., in dark condition. Dry seeds of the plant were subject to hydrodistillation using a Clevenger-type apparatus. The composition of essential oil was analyzed by gas chromatography mass spectrometry. The predominant components in the oil were linalool (57.57%) and geranyl acetate (15.09%). The mortality of 1–7-day-old adults of the insect pests increased with concentration from 43 to 357 **μ**L/L air and with exposure time from 3 to 24 h. In the probit analysis, LC_50_ values (lethal concentration for 50% mortality) showed that *C. maculatus* (LC_50_ = 1.34 **μ**L/L air) was more susceptible than *T. confusum* (LC_50_ = 318.02 **μ**L/L air) to seed essential oil of this plant. The essential oil of *C. sativum* can play an important role in stored grain protection and reduce the risks associated with the use of synthetic insecticides.

## 1. Introduction

The global pest-harvest grain losses by insect damage and other bioagents range from 10% to 40%. Methods used to control stored grain insect pest included physical, chemical, and biological treatments [1]. Chemicals largely used as pesticides in crop protection could have undesirable effects such as ozone depletion, environmental pollution, toxicity to nontarget organism, pest resistance, and pesticide residues, in addition to direct toxicity to users [1]. Therefore, the development of bio insecticides has been focused as viable pest control strategy in recent years [2, 3].

Plants may provide potential alternative to currently used insect-control agents because they constitute a rich source of bioactive chemicals [4]. Aromatic plants are among the most efficient insecticides of botanical origin and essential oils often constitute the bioactive fraction of plant extracts [3].

Essential oils are secondary metabolites abundant in aromatic plants families such as Lamiaceae and Apiaceae which contain a large number of compounds such as monoterpens and sesquiterpenes. Essential oils are known to exhibit low toxicity to mammals, and the most terpenoids and phenols found in plant essential oil have minimal toxicity and have even been approved as flavoring agents in food [2, 5]. The insecticidal [6, 7], nematicidal [8], and antibacterial [9] effects of coriander essential oil have previously been reported.

*Coriandrum sativum* (Apiaceae) is a native of the Mediterranean region and is grown in North Africa, central Europe, and Asia as a culinary herb and medicament. The essential oil of *C. sativum* exhibited volatile toxicity to stored product insects. López et al. [6] fractionated the seeds of *Coriander sativum* by column chromatography and tested them in the laboratory for volatile toxicity against three stored rice pests, *Sitophilus oryzae*, *Rhyzopertha dominica *and *Cryptolestes pusillus*. Their experiment showed the active compound of coriander essential oil against *S. oryzae* was linalool, while the fractions that contained mixtures of linalool, camphor, and geranyl acetate were as active against *R. dominica*, and *C. pusillus* as linalool alone. Also the results of Islam et al. [7] showed that the* C. sativum* essential oil had fumigant toxicity on* Tribolium castaneum* adults, larvae, and pupae.

The present study was carried out to determine the fumigant toxicity of essential oils extracted from seeds of *C. sativum* against adult stage of two other stored product insects, *Tribolium confusum* and *Callosobruchus maculatus*.

## 2. Materials and Methods

*Callosobruchus maculatus* and *Tribolium confusum* were reared in plastic containers (20 cm length, 14 cm diameter and 8 cm height) containing bean grain and wheat flour mixed with yeast (10 : 1, w/w), respectively, which were covered by a fine mesh cloth for ventilation. Adult insects, 1–7 days old, were used for fumigant toxicity. The culture was maintained in the dark in growth chamber set at 27 ± 1°C and 65 ± 5 relative humidity. All experiments were carried out under the same environmental conditions.

Seeds of *C. sativum* were collected in August, 2009, from Zabol region located in Sistan and Baluchestan province, Iran. The plant material was dried naturally on laboratory benches at room temperature (23-24°C) for 7 days until crisp. The dried materials were stored at −24°C and then hydrodistilled to extract its essential oil.

Essential oil was extracted from the plant samples using a Clevenger-type apparatus where the plant material is subjected to hydrodistillation. Conditions of extraction were fifty grams of seeds samples, 1 : 10 plant material/water volume ratio, and 4 h distillation. The *C. sativum* oil was dehydrated with anhydrous sodium sulphate and stored in airtight glassware in refrigerator at 4°C until being used in the treatment.

The essential oil of the *Coriandrum sativum* seeds was analyzed on a gas chromatograph (Varian suturn 2200) mass spectrometer (varian cp 3800) (GC-mass). The GC column was DB-5 (30 m × 0.25 mm i.d, 0.25 *μ*m film thickness). The GC conditions were as follows: injector temperature, isothermal at 50°C for 1 min, then programmed to 200°C at 4°C/min. Helium was used as the carried gas at the rate of 0.8 mL/min. The effluent at the GC column was introduced directly in to the source of the MS. Spectra were obtained in the EI mode with 70 eV ionization energy. The sector mass analyzer was set to scan from 40 to 300 amu for 1s. Unknown essential oil was identified by comparing its GC retention time to that of known compounds and by comparison of its mass spectra, either with known compounds or published spectra.

To determine the fumigant toxicity of the *C. sativum* oil, filter paper (2 cm diameter) was impregnated with oil at doses calculated to give equivalent fumigant concentration 43–357 *μ*L/L air. The impregnated filter papers were then attached to the screw tightly caps of glass with volumes of 70 mL. Caps were screwed tightly on the glass, each of which contained separately 10 adults (1–7 days old) of each species of insect. Each concentration and control was replicated four times. Mortality was determined 3 to 24 hours from commencement of exposure in serial time method. When no sign of leg or antennal movement was observed, insect was considered as dead.

Insect mortality percentage in another experiment was designed to assess 50% lethal doses (LC_50_). A series of dilutions was prepared to evaluate mortality of insect after an initial dose-setting experiment for determination concentrations with about 10 to 90% mortality. Concentrations of the oil were tested at 0.4, 0.8, 1.2, and 2 *μ*L/L air on *C. maculatus* and evaluated at 92.5, 185, 278, 370, and 462.5 *μ*L/L air on *T. confusum*, respectively. Control insects were kept under the same conditions without any essential oil. Each dose was replicated five times. The number of dead and live insects in each bottle was counted 24 h after initial exposure to the essential oil. The mortality was determined as described in previous experiment. The treatment bottles were monitored for at least 48 h after recording the data and no affected insect recovered. Data obtained from each dose response bioassay were subjected to probit analysis. LC_50_ values were estimated by probit analysis using SPSS 16.0.

## 3. Results and Discussion

Chemical composition of essential oil from the seed of coriander, *Coriandrum sativum*, is given in Table 1. The major components in the essential oil from *C*. *sativum* seeds were found: linalool (57.57%); geranyl acetate (15.9%); *β*-caryophyllene (3.26%), camphor (3.02%), and p-cymene (2.5%). Chemical analysis indicated clearly that linalool was the main component of coriander essential oil.

The result of our analysis was in agreement with the other literatures that reported linalool as major constituent in the essential oil of coriander [6, 10, 11]. It has been reported that major compounds in the seed coriander oil in Bangladesh were linalool (37.7%), geranyl acetate (17.6%), and *γ*-terpinene (14.4%) [11]. However, the difference in the oil composition was related to the relative proportion of the constituents and not to the presence/absence of a particular component. These variations may be attributed mainly to the plant part, the season (temperature, photoperiod, and hygrometry), the method of harvesting, the geographical zone, pedological condition, and the method used to isolate the plant product [12–14].

In all cases, considerable differences in mortality of insect to essential oil vapor were observed in different concentrations and exposure times. The mortality increased with rising concentrations from 43 to 357 *μ*L/L air and with exposure time from 3 to 24 h.

**Figure 1:**
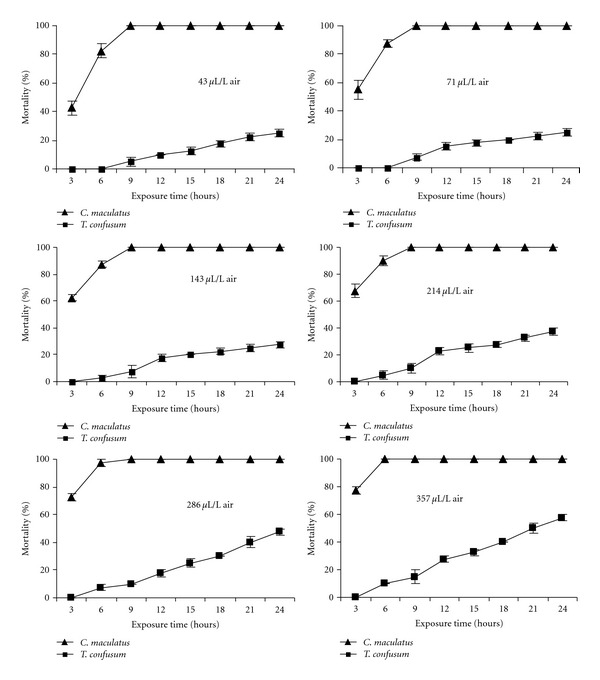
Cumulative percentage mortality of *Tribolium confusum* and *Callosobruchus maculatus* exposed to various concentrations of essential oils from *Coriandrum sativum* seeds at various periods of time.

Results indicated that the oil was relatively more toxic against *C. maculatus* than *T. confusum* (Figure 1). The lowest concentration (43 *μ*L/L air) of the oil yielded 100% mortality of *C. maculatus* after 24 h exposure but the mortality of *T. confusum* at the same concentration was 25% after 24 h.

The toxicity of *C. sativum* oil on *C. maculatus* and *T. confusum* was significantly different, as inferred by the confidence intervals of LC_50_ (Table 2). Probit analysis showed that *C. maculatus* was more susceptible (LC_50_ = 1.34 *μ*L/L air) to *C. sativum* oil than *T. confusum* (LC_50_ = 318.02 *μ*L/L air) (Table 2). A difference response of the insect species to the essential oils has previously been reported for stored product insects [15–17].

The insecticidal property of coriander essential oil was reported in previous studies and its toxicity generally was attributed to linalool [6, 7], the volatile compound which was found to be the primary component of seed oil in our chemical analysis. Moreover, the high toxicity of linalool and camphor was reported against stored products pests [16, 18, 19].

Insecticidal effects of *C. sativum* seed essential oil on adults of *Tribolium castaneum* and *Sitophilus oryzae*, *Rhyzopertha dominica,* and *Cryptolestes pusillus* have been shown before [6, 7]. Islam et al. [7] reported that fumigant LC_50_ value of *T. castaneum* at 24 exposure for adults insect was 0.011 *μ*g/mL acetone in glass vials (25 × 50 mm) or in other words nearly 448 *μ*g/L air. This value was more than the LC_50_ value calculated for *T. confusum* species (318 *μ*L/L air) in our study. More recently, it is shown that the seed oil of the *Coriandrum sativum* with linalool as major component (55.1%) has significant toxic effects against the larvae of *Aedes aegypti* with an LC_50_ value of 21.5 ppm and could play an important role as immunotoxicity on the insect [20]. The observed difference seems to be reasonable because of different insect species and methodology of oil extraction, as shown in similar experiments with various stored product pests and essential oil vapors [21, 22].

Studies on the mode of action of the natural insecticide have shown that treatments the insects with natural compounds such as essential oils or pure compounds may cause symptoms that indicate neurotoxic activity including hyperactivity, seizures, and tremors followed by paralysis (knock down), which are very similar to those produced by the insecticides pyrethroids [23]. It has been recognized that essential oils are potent neurotoxins and could affect through acetyl cholinesterase enzyme inhibition in the central nervous system [24]. 

Coriander oil and its major constituent, linalool, have low acute oral and dermal toxicity in laboratory animals. The acute lethality studies suggest that the toxicity of the oil is from its constituent, linalool. The acute oral LD_50_ of the major constituent of coriander oil, linalool, in Osborne Mendel rats was reported to be greater than 2.79 g/kg [25]. Collectively, these studies indicate that coriander oil and its major constituent, linalool, are of slight acute toxic potential [26]. As coriander oil contains approximately 70% linalool, the oral LD_50_ studies suggest that the lethality caused by the oil is from its constituent, linalool.

## 4. Conclusions

Results of the study show that the essential oil of *C. sativum* seeds can play an important role in stored grain protection and reduce the risks associated with the use of synthetic insecticides. The mode of action of *C. sativum* oil is of special interest. Further work should focus on its penetration into insect cuticle and grain, metabolic target in the insect body and, its effects on mammals fed on treated material. Therefore essential oil from *C. sativum* can become an interesting alternative to conventional chemical control strategies.

## Figures and Tables

**Table 1:** Chemical composition of the essential oil from *Coriandrum sativum *seeds.

Compounds^a^	Composition %	Kovats index
Heptanol	0.78	899
**α**-Thujene	0.19	928
**α**-Pinene	0.12	939
Sabinene	0.58	972
*β*-Pinene	0.14	979
l^3^-Carene	0.11	1005
**α**-Terpinene	0.39	1010
**p-Cymene **	**2.52**	**1028**
Limonene	0.62	1031
1,8-Cineol	0.97	1036
(z)-*β*-Ocimene	0.41	1040
*γ*-Terpinene	0.11	1062
Cis-linalool oxide	0.07	1070
Trans-linalool oxide	0.83	1080
Terpinolene	0.17	1092
**Linalool**	**57.57**	**1102**
*β*-Citronella	0.39	1127
**Camphor**	**3.02**	**1146**
**Borneol**	**1.27**	**1165**
Menthol	0.54	1170
Terpinene-4-ol	0.14	1177
p-Cymen-8-ol	0.29	1184
**α**-Terpineol	0.18	1188
Cis-Dihydrocarvone	0.35	1195
**Nerol**	**1.98**	**1226**
Neral	0.29	1237
**Carvone**	**1.14**	**1242**
Geraniol	0.24	1251
Geranial	1.03	1266
Anethole	0.21	1285
Thymol	0.12	1293
Carvacrol	0.29	1300
**δ**-Elemene	0.56	1340
Eugenol	0.76	1355
Neryle acetate	0.01	1363
**Geranyl acetate**	**15.09**	**1382**
***β*-Caryophyellene**	**3.26**	**1427**
**α**-Humulene	0.19	1454
Germacrene D	1.01	1484
Eugenyle acetate	0.49	1524
Other compounds	1.60	—

^a^Identification based on authentic standards, NIST library spectra, and literature.

**Table 2:** Efficiency of essential oil extracted from *Coriandrum sativum* seeds against *Tribolium confusum* and *Callosobruchus maculatus*.

Insects	No.*	LC_50_ (95% CL)	*χ* ^2^ (df)	Probability	Slope ± SE
*T. confusum *	250	318.02 (276.96–375.80)	3.93 (3)	0.269	2.85 ± 0.41
*C. maculatus *	250	1.34 (1.12–1.69)	1.80 (3)	0.614	2.06 ± 0.36

*Ten individuals per replicate, five replicates per concentration, and five concentrations per assay; LC: lethal concentration (*μ*L/liter air), CL: confidence limits, *χ* ^2^: Chi-square value, df: degrees of freedom.
